# Notes of Hospital Clinics

**Published:** 1897-10-02

**Authors:** 


### Notes of Hospital Clinics.

*[Reported for* The Hospital, *and corrected by the authors.]*

### ST. BARTHOLOMEW'S HOSPITAL.

### Clinical Lecture on Four Cases of Head Injury.

By Howard Marsh, F.R.C.S., Surgeon to the Hospital.

The cases of head injury which I have to relate were the following: The first was that of a boy aged eight, who shortly before admission was knocked down in the street, and kicked on the head by a horse. When he was brought to the hospital he had a wound three and three-quarter inches long in the right occipito-parietal region, in the neighbourhood of the sagittal suture, and at the bottom of this wound was a comminuted and depressed fracture of considerable extent. The boy was quite conscious, and there was no shock. After his head had been shaved, and the wound rendered as far as possible aseptic by prolonged scrubbing with soap and water, washing with ether, and by free swabbing with carbolic lotion (1 in 40)—he was removed to the operating theatre, and a full view of the extent and disposition of the fracture was obtained by enlarging the wound. It was then found possible to elevate one of the smaller fragments, and when this had been done the other depressed portions of the skull were raised into place. The dura mater was entire. The wound was now thoroughly douched with carbolic acid lotion and its torn edges pared with scissors, and then the fragments (which had been removed and which had been at once placed in a warm concentrated solution of boracic acid) were cut up into small pieces, and planted upon the dura mater. The wound was then closed. It healed by primary union, and the patient made an uninterrupted recovery.

The second case was that of a boy aged nine, who was admitted on June 4th with a small punctured wound of the scalp, about an inch above the left eyebrow. He had been struck with a stone thrown by another boy about 18 hours before his admission. He had for a time been unconscious, and had vomited repeatedly. On his admission he had recovered consciousness, and had no symptoms of any kind. On examination, however, it was found that a depressed fracture had occurred. When the wound had been carefully prepared in the manner described in the previous case, a crescentic flap was turned back and the fracture exposed. It was found to be of small extent (only an inch in its longest axis), but it was comminuted and very abruptly depressed. It was possible at one spot of the fracture to introduce an elevator, by which one of the fragments was removed. An opening was thus gained through which an instrument could be introduced, and the whole depression was elevated. The dura mater was entire. The wound was now copiously irrigated with carbolic lotion, and the bone replaced (after it had been cut into small fragments). The wound, after its edges had been pared, was closed over the fracture. Some superficial suppuration occurred, but this was no matter for surprise, as the wound had been inflicted 18 hours before any treatment was adopted. The boy, however, did quite well. The wound healed by granulation at the end of about ten days. No cerebral symptoms were developed.

Case 3: On July 3rd a boy, aged eight, was admitted with a long scalp wound in his occipito-parietal region, and beneath it an extensive comminuted and depressed fracture of the skull, produced by the kick of a horse, which had been received shortly before his admission. When first seen he was unconscious, and in a state of collapse. When the scalp wound had been prepared as in the other cases the fracture was exposed. It was extensively comminuted and deeply depressed. An elevator was inserted under the edge of one of the fragments, and this fragment raised. Very free hæmorrhage occurred, and when the other fragments had been elevated it was seen that the dura mater was extensively torn, and the subjacent brain considerably lacerated—almost, in fact, reduced to a pulp. Bleeding, as soon as the parts were exposed, ceased. The subjacent portion of the brain was thoroughly irrigated with warm carbolic lotion. The jagged edges of the wound were cut away with scissors, a drain of sterilised gauze was introduced, and the wound was closed around the drain, and dressed with cyanide gauze, and an ample covering of alembroth wool. For the first two days the boy remained in a drowsy condition, and was at times very restless, tossing himself about, and trying to get out of bed—symptoms which indicated cerebral irritation. His temperature, however, remained normal, the wound healed favourably, and he made a very good recovery.

As you are aware, it is laid down by all surgical writers of the present day that in cases of compound depressed fracture of the skull, whether there are symptoms or whether there are not, the patient must be immediately trephined. This expression is not quite an accurate one. The real meaning of the rule is that operative interference to elevate the depressed fragments must be invariably adopted. In many cases, however, there is no necessity to trephine, for elevation of the fragments can be effected without this. I do not wish to convey the idea that trephining involves any increase of danger to the patient, but I think you will often find in practice, especially when you are dealing with young subjects, that you will be able to insert your elevator at some point between the fragments, and thus to raise them into place. Obviously, you must be extremely careful that in trying to insert your elevator you do not drive the fragments in, or make one of them a fulcrum against which to rest your instrument. If in any case you find the fragments are closely impacted you ought—using quite a small trephine—to cut out a crown from the sound bone at the edge of the fracture, and thus gain access to the depressed fragments. You always operate in order to prevent further brain injury by the depressed fragments. But in the present day there is a second and very strong reason for operating, which you must fully realise. You must remember that you are dealing with a compound fracture, and that the wound, in all probability, is septic. In this case, as in an ordinary compound fracture of the leg, for instance, all parts of the wound must be fully exposed and rendered as completely as possible aseptic. This, obviously, can only be done when the fragments are all elevated, and access is gained to the dura mater, or to the substance of the brain if the latter is exposed.

Case 4: A man aged 52, while in a state of intoxication on Easter Monday, was thrown out of a cart upon his head. When brought to the hospital he was found to have sustained a scalp wound, about two inches long, in the neighbourhood of the right parietal eminence. No fracture could be detected, and he had no symptoms besides those which might be due to drink. He was, however, detained for eight or ten hours, and was then allowed to go home. Next day his friends found that he was quite unable to speak. Thirty-six hours afterwards he was noticed to have some twitching of the muscles of the mouth on the right side, and a few hours later convulsive movement of the right hand and forearm. He was now brought to the hospital again. We found that he was in a state of complete aphasia, and convulsions involving the right side of the face and the right upper extremity took place every four or five hours. These symptoms seemed clearly to indicate that there must be a blood clot situated in the neighbourhood of Broca's convolution, and the motor area for the face and upper limb. On the following day, therefore, I trephined over a spot a little in front of the lower extremity of the fissure of Rolando. The dura mater was entire, but it protruded into the opening, and no pulsation of the subjacent brain could be felt. On opening the dura mater we came down upon a blood clot about as large as half-a-crown, and about a quarter of an inch in thickness at its centre. This was carefully removed. The brain beneath it bore marks of contusion. The dura mater was replaced and sutured. The trephine crown, which had been placed in warm boracic solution, was cut into small fragments, and planted in the opening, except at the centre, where a space was left for the escape of any exudation that might occur. For the next three days the symptoms remained very much the same, but after this date the convulsive movements slowly subsided, and a week later the patient began to speak again. His improvement was slow but continuous, and at the end of a fortnight all the convulsions had ceased, and his aphasia had almost disappeared. He ultimately made a very good recovery.

These cases may serve to illustrate the different forms of evidence upon which you will have to depend in making a diagnosis in different instances. As you know, there are two chief forms of evidence—the direct or positive, and the indirect or circumstantial. In the first three cases I have related the evidence was positive, for the fracture of the skull could be absolutely seen, and its features in each case were matters of eyesight and touch; it could be both seen and felt that the fractures were comminuted and depressed. Now, this positive form of evidence very often amounts to absolute proof, so that all doubt is completely abolished. You must, however, remember that evidence of a positive kind may, unless you take great care, lead you into error. You observe positive facts, but these facts do not bear the construction which you put upon them. Let me give you an illustration. During an engagement a soldier was seen lying on the ground bleeding and in a state of shock, and the end of a femur was seen to be projecting through a lacerated wound in the front of his thigh. Now, is it not clear from this positive evidence that this patient had sustained a compound fracture of the thigh with projection of one end of the bone? This would seem a perfectly obvious conclusion from the facts. On examination, however, it was found that he had not sustained a fracture of his thigh, but that the bone which was seen in the wound was that of another soldier; it had, in fact, been driven out of the thigh of one man and impaled in the thigh of another—a very extraordinary accident no doubt, but one that has been recorded in the annals of military surgery. You thus see that even positive evidence may be deceptive, and that in dealing with it you must still exercise constant care and critical thought.

It is notorious in the law that circumstantial evidence is likely to be very deceptive, and many illustrations of this could easily be given. It has often been pointed out, however, that circumstantial is sometimes an exceedingly strong form of evidence—quite as strong as any positive evidence can be. Circumstantial evidence is thus strong when a large number of circumstances point in the same direction, and in which it seems impossible to explain these except by some particular conclusion. You will notice that the diagnosis of the last case was entirely circumstantial. The circumstances were: (1) The slow development of aphasia after an injury to the head; (2) the development a day or two later of muscular twitchings on the right side of the face; and (3) a little later still, the onset of convulsive movements of the right hand and arm. These items of circumstantial evidence, taken altogether, conveyed an extremely strong probability that a clot had formed in a definite position, *i.e.*, in the immediate neighbourhood of Broca's convolution, and over the motor area of the face and upper limb. The slow development of the symptoms was a circumstance that seemed to point clearly to the presence of a clot rather than to any other condition. In this case circumstantial evidence guided us to a correct conclusion. But I want emphatically to say that you must, just as the lawyers do, accept circumstantial evidence with very great caution. When, as must often be the case, you have to resort to it, be very careful to collect every circumstance connected with the patient's illness, and take all these circumstances into consideration. Think them all over with most diligent care, and form your diagnosis only after you have as far as possible balanced all the probabilities.

